# Technique alternatives for breast radiation oncology: Conventional radiation therapy to tomotherapy

**DOI:** 10.4103/0971-6203.54849

**Published:** 2009

**Authors:** N. Fournier-Bidoz, Y. Kirova, F. Campana, J. El Barouky, S. Zefkili, R. Dendale, M. A. Bollet, A. Mazal, A. Fourquet

**Affiliations:** Department of Radiation Oncology, Institut Curie, 26 rue d'Ulm, Paris 75005, France

**Keywords:** Breast, intensity-modulated radiation therapy, radiation oncology

## Abstract

Breast conserving radiotherapy uses tangential fields and compensating wedges. This conventional approach can be improved by a field-in-field technique using the linac multi-leaf collimator (MLC). A simplified field-in-field technique that planners can easily achieve and which improves dose uniformity in the breast volume is presented here. Field junction problems are more easily solved by the use of a virtual simulation. A unique isocenter can be set at the junction between the supra-clavicular field and the breast tangential fields. However, careful quality assurance of the treatment planning system must be performed. Tomotherapy has promising clinical advantages: the ability of a tomographic image to correct for random set-up errors, a continuous cranio-caudal delivery which suppresses junction problems, the conformality of the dose distribution throughout the complex volumes formed by the lymph nodes and the breasts. Tomotherapy is a valuable recourse for complex irradiations like bilateral breast or mammary plus axillary irradiation while a field-in-field associated with a unique isocenter technique can be used for majority of the patients.

## Introduction

Breast cancer treatments represent 30 to 40% of the activity of a Radiation Oncology department. A change of radiation technique is therefore of consequence and necessitates new organization, teaching, and resources. New features have recently been implemented in treatment planning systems (TPS) and treatment machines which allow a better dose distribution, more accurate dose calculations and easy radiation delivery.

## Materials and Methods

Breast conserving radiotherapy uses tangential fields. Any other beam incidence would lead to useless irradiation of the underlying lung and heart. Minimum standards for breast radiotherapy include the use of solid or dynamic wedges for a better dose uniformity. Calculations account for lung density and are performed on at least, three CT-slices or contours so that a field reduction can be planned to avoid over-dosage in the inferior breast.

### Breast compensation techniques

**Figure 1:**
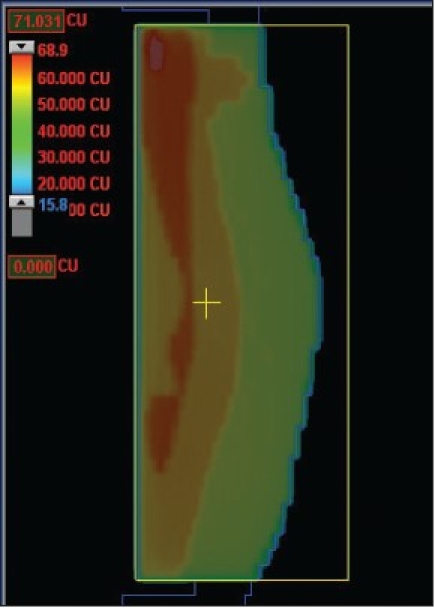
Fluence for breast surface compensation using simplified IMRT with dynamic leave delivery

Wedges cannot compensate for a change in breast shape in the cranio-caudal direction. A field reduction is necessary at the breast fold to avoid over-dosage and treatment complications in this area. Dose uniformity throughout the whole breast volume can be achieved by using multi-leaf collimator (MLC) sub-fields; these are shaped to the successive iso-doses that are found in the dose distribution before compensation.[1] This is a forward planning approach which is not based on planning target volume (PTV) delineation. An alternative is the use of dynamic leaves to produce intensity-modulated fields. This technique, also called simplified intensity-modulated radiation therapy (IMRT), is based on the calculation of a set of transmission factors with a typically 5×2.5 mm^2^ resolution to get 100% of the prescribed dose at mid-separation at all beamlets' incidences. It is able to compensate for the complex external shape of the breast and produces a homogeneous dose distribution. When looking at the resulting intensity fluence, it appears that there are mainly three ranges of transmission factors [Figure 1]. In other words, one would need a three layer transmission block, one layer being of thickness zero, to achieve the best homogeneity in the breast. Quality assurance must be performed to ensure that the TPS calculation is close enough to the actual delivery accounting for leaves transmission, speed and shape.

### Simplified field-in-field technique

**Figure 2:**
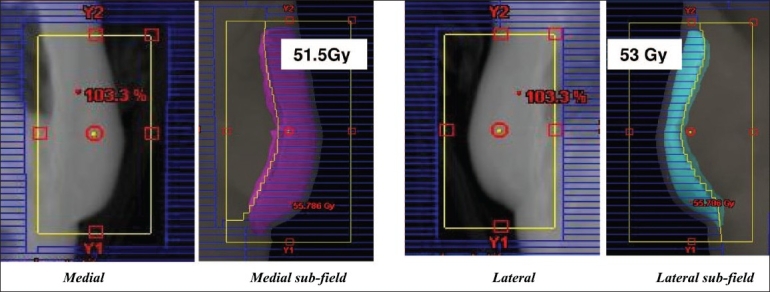
Medial field and sub-field BEV with 51.5 Gy iso-dose display; Lateral field and subfield BEV with 53Gy iso-dose display. Maximum dose in the volume is 55 Gy (110%) before inclusion of subfields

**Figure 3:**
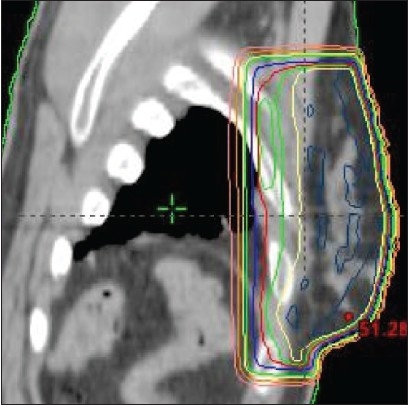
Sagittal dose distribution after adjustment of subfields weights: green iso-dose is 47.5 Gy (95%), yellow isodose is 49 Gy, maximum dose is 51 Gy

At our Institute a field-in-field technique, that is easily achieved by the dosimetrists and applied to all breast patients treated at a linac with MLC, is used. The breast limits are determined by the physician by clinical palpation and marked by radio-opaque markers for CT acquisition. Two open photon tangents are set on the virtual simulation and dose distribution is calculated. The resulting plan is normalized at mid-separation between the two tangents entry points at central axis plane. Fifty grays are prescribed to that point. Iso-dose lines from 48 Gy to 53 Gy by step of 0.5 Gy are visualized on the sagittal plane. Two iso-dose lines which are significantly large and well separate from each other are chosen. The TPS (Eclipse, Varian) is able to show the 3D outline of each iso-dose projected on the tangent beam's eye view (BEV). As an example, Figure 2 shows the 51.5 Gy and 53 Gy iso-doses on the medial and lateral fields respectively. For each tangent one sub-field is created with the MLC shaped to one of the two iso-doses. Weights are subsequently adjusted so that the dose distribution is uniform within the PTV, as recommended by the ICRU guidelines (95% to 107%) at all levels in the breast [Figure 3]. The sub-field weights generally represent a four to eight per cent fraction of the treatment time for one field. We have fixed a minimum treatment time at 10 monitor units (MU). If the uniformity is obtained for less than 10 MU, one of the sub-fields is suppressed.

**Figure 4:**
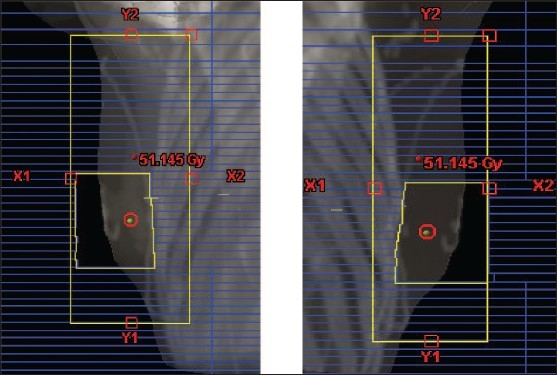
BEV of concomitant boost sub-fields

An important advantage of this technique is that a concomitant boost can be integrated as a second field-in-field within each tangential beam [Figure 4]. The boost sub-field weights are adjusted to give the prescribed dose and fractionation to the tumor bed volume.

Treatment delivery is performed in a “step-and-shoot” automatic mode by the linac.

Quality assurance was done at TPS commissioning to check the accuracy of the TPS dose calculation and the step-and-shoot delivery using dosimetric films before releasing the technique into clinical use. Main discrepancies were found at leave edges and beam corners but were within acceptable levels (three per cent, three mm).

This forward planning technique has several advantages: breast volume is localized clinically; planning is rapidly and easily performed by the dosimetrists; it requires no special quality assurance (QA). The technique was then applied to all patients treated on a linac with MLC. Moreover, a concomitant boost sub-field can be designed using either a conventional approach (the radiation oncologist deciding for the field borders) or a tumor bed CTV drawn by the radiation oncologist.

### Field junctions

**Figure 5:**
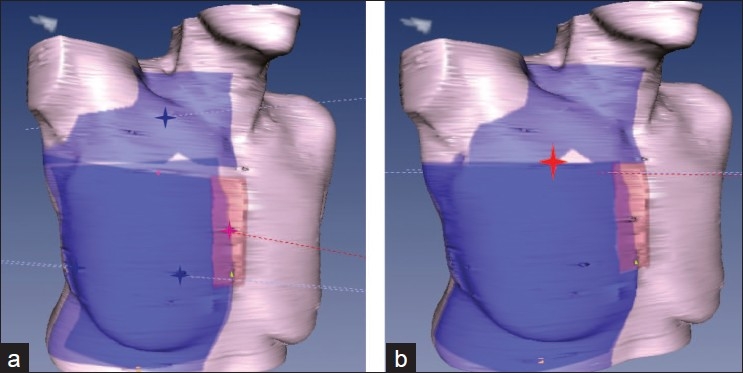
a) Multi-isocenter b) Unique isocenter for supra-clavicular, internal mammary chain, and breast field set-up

Field junction dosimetric problems are more easily solved by the use of a virtual simulation. Multiple adjacent fields lead to hot and cold spots at the field junctions when treating the lymph nodes surrounding the breast [Figure 5a]. A commonly used way to overcome this problem is to use asymmetric jaws to create a half beam for supra-clavicular and IMN fields and, by couch rotations, to align the superior edge of the tangents to the inferior edge of the supra-clavicar field. This adds complexity for the technologists during patient set-up. Moreover, treatment couch must be moved to go from one beam to the other in order to treat each volume. A unique isocenter can be set at the junction between the supra-clavicular field and the breast tangents.[2] The supra-clavicular, internal mammary fields and tangents are set as half beams (use of asymmetric jaws) so that there is no divergence at the junction [Figure 5b]. Dose points placed in each volume serve as normalization and prescription points.

### Quality Assurance

The planning procedure should be written and verified before clinical use. The TPS ability to calculate the dose distribution and treatment times according to the normalization and weights, MLC shapes and transmission, merging of sub-fields for treatment delivery must be checked by measurements before releasing the technique into routine clinical use. The TPS capability of accurately calculating dose distributions in complex geometries with large asymmetries like in the mono-isocenter technique must be verified according to International recommendations.[3]

Dosimetric films can be used to compare TPS calculations and field-in-field plans delivery. Radiochromic films are easy to use as they do not need film processing. Figure 6a shows a Gafchromic film irradiated at 5cm in a flat phantom to verify the calculated dose of a breast lateral tangent containing one subfield and having an asymmetric geometry. The dose distribution in the phantom in a frontal plane is compared to the dose distribution on the film [Figure 6b].

**Figure 6:**
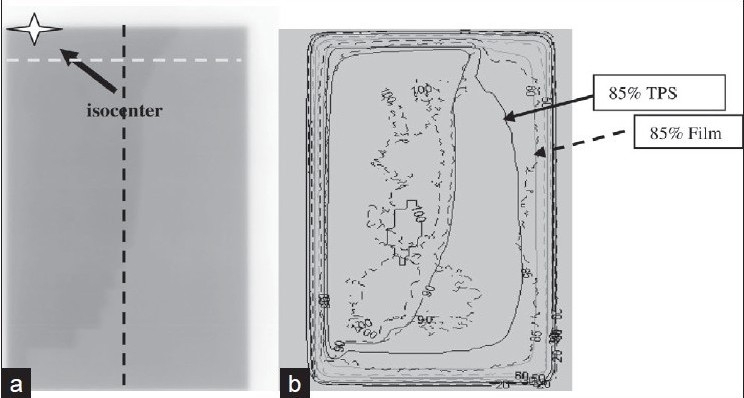
a) Dosimetric film for a two-segment asymmetric breast field b) Superposition of calculated (plain) and measured (dashed) iso-doses

The calculated iso-dose 90% fits well with the measured 90% [Figure 6b]. However, discrepancies are found at the corners of the asymmetric field furthest from the isocenter -85% iso-dose on film does not thus agree with the calculated 85% [Figure 6b]. Two profiles [dashed lines on Figure 6a] are then analized [Figure 6c]. The measured profile dose is higher than the calculated dose. The shoulders of the calculated profiles are more rounded than the measured ones. Even though the mean dose difference remains within three per cent, it is relevant to come back to the TPS beam data base and look at the accuracy of large fields and MLC edges and transmissions modelization.

**Figure 6c:**
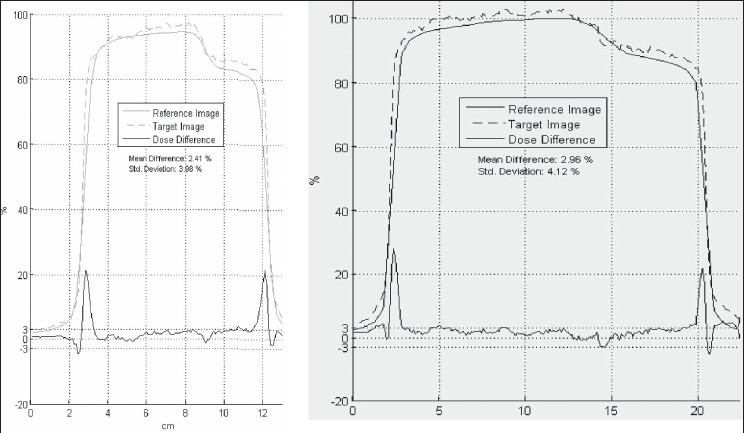
Calculated (plain) and measured (dashed) profiles along the two orthogonal lines. Mean difference is two to three per cent (Std deviation four per cent)

### Inverse planning with tomotherapy

The advantage of using intensity modulated radiation therapy (IMRT) in breast radiation oncology stays controversial. There are geometric uncertainties in the actual borders of the mammary glandular tissue and of lymph node volumes.[4] Most radiation oncologists would rather rely on a clinical localization of the breast limits than on CT images of the mammary tissue. Organ at risk sparing (mainly lungs and heart) is conventionally defined by the values of MHD (maximum heart distance) and CLD (central lung distance) as read on tangent fields radiographies. These quantities have been shown to be related to the volumetric dose of heart and lung.[5] The long acquired clinical experience with tangent fields must therefore be put aside when using IMRT with multiple beam directions like in helical tomotherapy. Tomotherapy has, however, some promising clinical advantages: the ability of a tomographic image to correct for set-up errors, a continuous cranio-caudal delivery which suppresses junction problems, the conformality of the dose distribution throughout the complex volumes formed by the lymph nodes and the breast.[6] Our institute has a tomotherapy machine (Tomotherapy Inc., Madison, USA) since 2007. Mainly head-and-neck and lung cancers have been treated with this machine. We are now evaluating the advantages of using tomotherapy in some complex breast cases.

We have performed the optimization of a tomotherapy treatment plan for a patient with bilateral breast cancers and positive nodes on the right side. Left target volume was limited to the left breast. Right targets were breast, internal mammary and supra-clavicular lymphs nodes. A virtual blocking structure was created to avoid irradiation from posterior incidences. The resulting dose homogeneity was excellent and conformality of the prescribed dose (50Gy to the breasts and 46Gy to the nodes) to the target volumes was obtained to the level V95 equal to 95%. Low doses to heart and lungs were obtained: V20 less than 20% for heart and lungs.

Quality assurance is performed using the tomotherapy phantom and an integrated QA module to project the plan intensities on the phantom. Two absolute dose point measurements are done with ionization chamber and a film placed in the phantom gives the relative distribution in one plane that are compared to the tomotherapy plan calculation.

## Conclusions

Progress in treatment planning software and treatment machines is leading to considerable progress in breast radiation therapy. A practical option must be chosen so that: a majority of patients will benefit from the progress in technology, changes are built on clinical experience, QA is compatible with the department workload. Our Institute field-in-field technique meets all these requirements. It improves the homogeneity of the dose distribution with the clinical aim of reducing skin toxicity. A concomitant boost can also be safely added without using inverse planning. Junction problems can be solved by the use of a unique isocenter when treating breast and surrounding lymph nodes. However, special care must be taken before clinical use to make sure that the TPS is giving accurate dose distributions and treatment times. An independent MU calculation program should be used in routine. Breast tomotherapy needs human resources along the preparation process: contouring of all target and organ at risk volumes, dosimetry optimization, quality controls. However, it is a valuable recourse for complex irradiations such as bilateral breast cancers or indications of breast and axillary irradiations.

## References

[1] Vicini FA, Sharpe M, Kestin L, Martinez A, Mitchell CK, Wallace MF (2002). Optimizing breast cancer treatment efficacy with intensity-modulated radiotherapy. Int J Radiat Oncol Biol Phys.

[2] Klein EE, Taylor M, Michaletz-Lorenz M, Zoeller D, Umfleet W (1994). A mono isocentric technique for breast and regional nodal therapy using dual asymmetric jaws. Int J Radiat Oncol Biol Phys.

[3] International Atomic Energy Agency (2004). Commissioning and Quality Assurance of Computerized Planning Systems for Radiation Tretament of Cancer. Technical Reports Series.

[4] (2003). BIR Working Party.

[5] Kong FM, Klein EE, Bradley JD, Mansur DB, Taylor ME, Perez CA (2002). The impact of central lung distance, maximal heart distance, and radiationtechnique on the volumetric dose of the lung and heart for intact breast radiation. Int J Radiat Oncol Biol Phys.

[6] Gonzalez VJ, Buchholz DJ, Langen KM, Olivera GH, Chauhan B, Meeks SL (2006). Evaluation of two tomo-therapy-based techniques for the delivery of whole-breast intensity-modulated radiation therapy. Int J Radiat Oncol Biol Phys.

